# A Chinese Patent Medicine JiaYanKangTai Alleviates Inflammatory Lesions of Experimental Autoimmune Thyroiditis by Regulating Interleukin-17 Signaling

**DOI:** 10.3389/fendo.2021.794568

**Published:** 2022-01-31

**Authors:** Yajing Pan, Qiue Zhang, Chengfei Zhang, Lili Wu, Lingling Qin, Tonghua Liu, Kaiwen Hu

**Affiliations:** ^1^ DongFang Hospital, Beijing University of Chinese Medicine, Beijing, China; ^2^ Key Laboratory of Health Cultivation of Beijing, Beijing University of Chinese Medicine, Beijing, China; ^3^ The First School of Clinical Medicine, Shaanxi University of Chinese Medicine, Xi’an, China

**Keywords:** JiaYanKangTai, Chinese medicine, autoimmune thyroiditis, interleukin-17, inflammatory lesions

## Abstract

**Objective:**

This study was aimed to investigate the effects of JiaYanKangTai (JYKT) on regulating interleukin-17 (IL-17) signaling in rats with autoimmune thyroiditis.

**Methods:**

Lewis rats were administrated with JYKT for eight weeks after a seven-week subcutaneous injection of thyroglobulin with adjuvant and feeding iodine water. Ultrasonography was performed and total volume of thyroid was calculated. The expressions of autoantibodies and hormones were detected. Morphological changes of thyroid were observed. Metabolomics profile and metabolic network analysis were conducted. IL-17 signaling was detected by polymerase chain reaction and immunohistochemistry separately.

**Results:**

JYKT reduced the mean volumes of thyroid, decreased both levels of TPOAb and TGAb, and alleviated lymphocytic infiltration of the thyroid. Metabolic network analysis of metabolomics proved IL-17 signaling pathway as a critical pathway in JYKT administration for autoimmune thyroiditis. JYKT downregulated expressions of IL-17A, TRAF6, p-ERK1/2 and TNF-α.

**Conclusion:**

JYKT alleviated inflammatory lesions of experimental autoimmune thyroiditis by regulating IL-17 signaling.

## Introduction

Autoimmune thyroid diseases (AITDs) are a class of the most frequent organ-specific autoimmune diseases with a high genetic heritability of 55-75%, and the main causes of thyroid dysfunction clinically (1, 2). The prevalence of AITDs differs with races, ages, genders and geographic regions with a morbidity of 5-15% in women and 1-5% in men (2, 3). Genetic susceptibility, environmental factors, some drugs, infection, psychologic stress are potential factors of the initiation and progression of AITDs (4). The pathologic hallmarks of AITDs are T-cell mediated immune responses and lymphocytic infiltration in the thyroid, leading to follicular destruction, gradual atrophy and thyroid fibrosis (3). Graves’ disease and Hashimoto’s thyroiditis (HT) are two commonest AITDs (5). Clinical symptoms of AITDs are diverse. AITDs affect not only the thyroid. Extrathyroidal symptoms include weight change, anxiety, general fatigue, malaise, palpitations, and so forth, affecting normal functions of the eyes, the brain and the heart, resulting in a decrease in the quality of life (3, 6). Proper therapy for AITDs is of great significance.

Some randomized controlled trials reported that Traditional Chinese medicine (TCM) is an effective and alternative therapy for AITDs (7–9). TCM is reported to be effective on relieving symptoms, decreasing levels of thyroid antibodies, alleviating follicular destruction and improving the quality of life with few side effects (10–12). JiaYanKangTai (JYKT) is a Chinese patent medicine, numbered ZL 2016 1 0642433.6, which is composed of 10 g of Bupleuri Radix, 20 g of Curcumae Radix, 30 g of Prunellae Spica, 15 g of Mume Fructus, 15 g of Fritillariae Thunbergii Bulbus, 10 g of Scrophulariae Radix, 10 g of Dioscoreae Nipponicae Rhizoma, 6 g of Cremastrae Pseudobulbus and 30 g of Astragali Radix (13, 14). Our previous clinical study showed that JYKT administration relieved symptoms, reduced the sizes of thyroid lobes and decreased autoimmune antibody levels in patients with Hashimoto’s thyroiditis (13). In addition, animal studies proved that JYKT decreased the levels of anti-thyroid peroxidase antibody (TPOAb) and anti-thyroid globulin antibody (TGAb), and regulated immune function in rats with autoimmune thyroiditis (14). However, the mechanisms of JYKT for autoimmune thyroiditis are not totally elucidated. This study was aimed to investigate the effects of JYKT on regulating interleukin-17 (IL-17), the major effector cytokine of Th17 cells, in autoimmune thyoiditis.

## Materials and Methods

### Reagents

Porcine thyroglobulin (pTg, T1126-1G), complete Freund’s adjuvant (CFA, F5881) and incomplete Freund’s adjuvant (IFA, F5506) were supplied by Sigma Aldrich (CA, United States). Anti-TPOAb ELISA kits (CSB-E11199r), thyroid stimulating hormone (TSH) ELISA kits (CSB-E05115r), free thyroxine (FT4) ELISA kits (CSB-E05079r), thyroxine (T4) ELISA kits (CSB-E05082r), free triiodothyronine (FT3) ELISA kits (CSB-E05076r) and triiodothyronine (T3) ELISA kits (CSB-E05085r) were supplied by Cusabio Technology (Wuhan, China). Taq Pro universal SYBR qPCR master mix (Q712) and HiScript III RT superMix for PCR were supplied by Vazyme (Nanjing, China). Antibodies to IL-17A (abcam150719), tumor necrosis factor receptor-associated factor 6 (TRAF6, ab33915) and tumor necrosis factor α (TNF-α, ab6671) were supplied by Abcam (Cambridge, MA, USA). Antibody to phospho-extracellular-signal-regulated kinase 1/2 (p-ERK1/2, 4370S) was supplied by CST (Danvers, MA, USA). JiaYanKangTai granules were supplied by Tcmages Pharmaceutical Co., Ltd (Beijing, China). Kits for aspartate aminotransferase (AST, AUZ8893), alanine aminotransferase (ALT, AUZ9151), creatinine (2598) and urea nitrogen (AUZ9114) were supplied by Beckman Coulter (Brea, CA, USA).

### Animals and Experimental Protocols

Six-week-old specific pathogen-free (SPF) level female Lewis rats [animal license number: SCXK (Jing) 2016-0006] with body weights of 120-150 g were obtained from Beijing Vital River Laboratory Animal Technology Co., Ltd (Beijing, China). Rats were housed under SPF conditions in a temperature-controlled room with a 12 h:12 h light-dark cycle in the laboratory of experimental animal center of Beijing University of Chinese Medicine [laboratory license number: SYXK (Jing) 2020-0033]. All procedures were approved by the Animal Care and Use Committee of Beijing University of Chinese Medicine (approval number: BUCM-4-202922901-4148).

**Figure 1:**
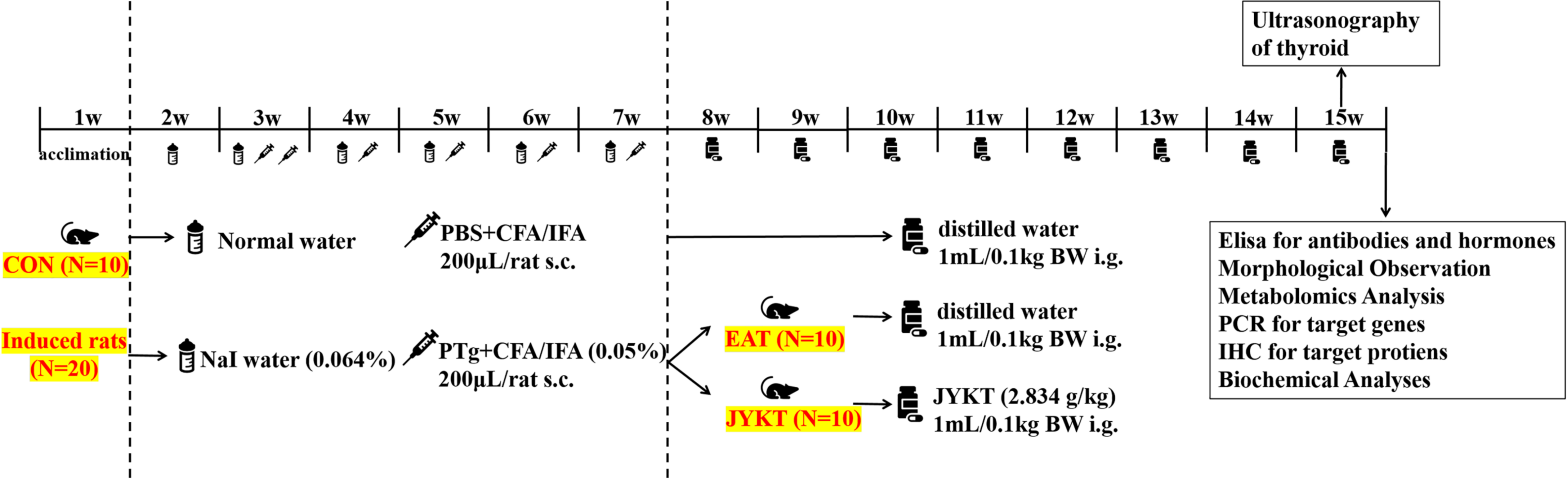
Experimental design.

After acclimation, thirty rats were randomly assigned into the control group (CON, n=10) and the induced group (n=20) using a computer-generated random numbers table. Ten rats of the CON group were provided with ad libitum access to food and water, and twenty rats of the induced group were treated with 0.064% sodium iodide in drinking water. After one week, experimental autoimmune thyroiditis (EAT) was induced in twenty rats of the induced group by immunization with thyroglobulin and Freund’s adjuvant, as described previously (14). Briefly, 0.1 mg PTg was dissolved in 100μL phosphate-buffered saline (PBS) and then emulsified with 100μL CFA (or IFA) to make the final concentration to be 0.05%. In the third week, 200μL freshly-prepared PTg with CFA was injected subcutaneously twice with an interval of two days at the back and groins to induce immunization. In the next four weeks, 200μL freshly-prepared PTg with IFA was injected once a week to boost immunization. Ten rats in the CON group were injected with PBS in the same way. One week after the final injection, blood was collected *via* the retro-orbital plexus to determine the levels of TPOAb and TGAb. Rats were then randomly divided into the EAT group (EAT, n=10) and the JYKT group (JYKT, n=10) according to the levels of autoantibodies. Daily oral administration of JYKT at a dosage of 2.834 g/kg per day were conducted in the JYKT group in the next eight weeks, while double distilled water was conducted in the same way in the CON group and the EAT group. The dosage of JYKT was determined based on the previous studies (14). The overall experimental design was shown in **Figure 1**.

### Ultrasonography of Thyroid

**Figure 2:**
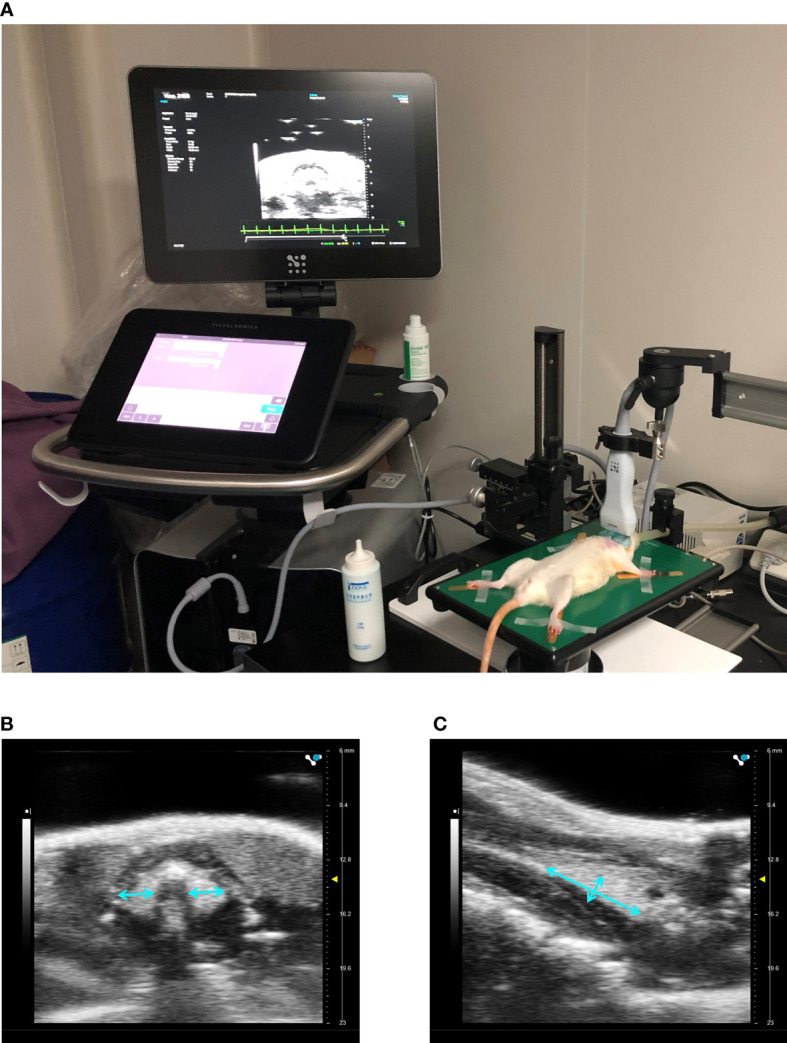
Ultrasonography of the thyroid. Preparation and setup of a rat on the micro-imaging system **(A)**. Imaging in transverse **(B)** and sagittal planes **(C)** of the thyroid.

Three days before sacrifice, ultrasonography of the thyroid was performed by a Vevo 3100 high-resolution micro-imaging system (VisualSonic, Toronto, Canada). The preparation and setup of ultrasonography was shown in **Figure 2**. Rats were anesthetized with 5% isoflurane at an oxygen flow rate of 2 L/min and maintained at 2% isoflurane throughout the procedure. Rats were placed in a supine position on the operating platform after hair of anterior neck being shaved. A transducer was coupled to the neck skin with coupling gel. The structure of thyroid was observed and the diameters were determined from craniocaudal, mediolateral and anteroposterior directions by an experienced technician. The volume of each lobe was calculated using the ellipsoid formula below (15) and the total thyroid volume was calculated by summing the volume of each lobe:


volume=π/6×craniocaudal×mediolateral×anteroposterior diameter

### Enzyme-Linked Immunosorbent Assay (ELISA)

Rats were sacrificed after anesthesia. Blood was collected *via* abdominal aorta and serum was separated by centrifugation at 3000 rpm for 10 min. Serum levels of TPOAb, TGAb, TSH, T4, FT4, T3 and FT3 were determined using ELISA kits according to the manufacturer’s instructions. Colorimetric measurement was conducted using a GloMax-Multi microplate reader (Promega, Madison, WI, USA) at an optical density of 450 nm. Standard curve was made and the concentrations were calculated by four-parameter logistic curve-fit.

### Morphological Observation

Thyroids of rats were harvested carefully before fixed with 4% formaldehyde for 48 hours, embedded in paraffin, and then cut into 3-µm-thick sections. Subsequently, parts of the sections were deparaffinized and rehydrated before stained with hematoxylin and eosin (H&E). Morphological changes of thyroid were observed by an experienced technician using a BX53F optical microscope (Olympus, Tokyo, Japan) at 200× magnification. The percentage of lymphocytic infiltration area was analyzed using Image-Pro Plus 6.0 software (Media Cybernetics, Silver Spring, MD, USA) (16, 17).

### Metabolomics Analysis

Serum was collected as mentioned above. 50 μL serum was mixed with 200 μL mixture of methanol and acetonitrile (v/v=1:1) containing isotopically-labelled internal standard before sonicated in ice water bath for 10 minutes and incubated in −20°C for 1 hour. The supernatant was separated by centrifugation at 12000 rpm at 4°C for 15 min. The supernatant as well as quality control was subjected to metabolomics profile analysis using a Vanquish ultra-high-performance liquid chromatography system (Thermo Fisher Scientific, Waltham, MA, USA) coupled with a Q Exactive HF-X Hybrid Quadrupole-Orbitrap mass spectrometer (Thermo Fisher Scientific, Waltham, MA, USA) platform by Biotree Ltd. (Shanghai, China).

After raw data being transformed to mzXML format using ProteoWizard, peak identification, peak extraction, peak alignment, and peak integration were performed using XCMS software. Orthogonal partial least squares discriminant analysis (OPLS-DA) was performed using SIMCA 11.0 software (Umetrics, Umea, Sweden). Metabolites with VIP>1 and *P*<0.05 were considered as differential metabolites and the heatmap was performed. After analyzed data being matched with the KEGG database, pathway enrichment and network analysis among groups were combined. Statistical analysis of metabolomics was performed using R 3.5.1 software (R Foundation for Statistical Computing, Vienna, Austria).

### Polymerase Chain Reaction (PCR)

Thyroids were gently dissociated and then stored at -80°C. Total ribonucleic acid (RNA) was extracted with TRIzol reagent. The concentrations of RNA were determined by measuring absorbance at 260 nm using a UV5 Nano spectrophotometer (Mettler Tolero, Switzerland). Complementary deoxyribonucleic acid (cDNA) was prepared with a HiScript III RT SuperMix reverse transcriptase kit by a T100 thermal cycler system (Bio-Rad, Hercules, CA, USA). Quantitative reverse transcription polymerase chain reaction was conducted with a Taq Pro universal SYBR qPCR master mix by an ABI 7500 real-time PCR system (Applied Biosystems, Waltham, MA, USA). Rat-specific PCR primers were designed. The sequences were as follows: *Il-17a*, forward 5’-CTC AAC CGT TCC ACT TCA CC-3’, reverse 5’-CAC TTC TCA GGC TCC CTC TTC-3’; *Traf6*, forward 5’-CCC AAG AAG AGG AAA GAC-3’, reverse 5’-AGG ATC GTG AGG CGT AT-3’; *Erk1*, forward 5’-CTG GCA CTG AAG GAG G-3’, reverse 5’-AAC AAG ATG AGG CTA CG; *Erk2*, forward 5’-CCA GAG TGG CTA TCA AGA AG-3’, reverse 5’-GGA TGT CTC GGA TGC CTA; *Tnf-α*, forward 5’-CCA CCA CGC TCT TCT GTC T-3’, reverse 5’-GCT ACG GGC TTG TCA CTC G-3’; *Gapdh*, forward 5’-CTC TGC TCC TCC CTG TTC-3’, reverse 5’-CGA TAC GGC CAA ATC C-3’. *Gapdh* was used as an endogenous control. The PCR results were expressed as the relative expression ratio of targeted genes and *Gapdh*.

### Immunohistochemistry (IHC)

Sections of thyroid were deparaffinized and rehydrated as mentioned above. Antigen retrieval was performed by boiling the sections in 10 mM sodium citrate buffer (pH 6.0) for 12 min. Sections were then blocked with ovalbumin and goat serum for 30 min respectively before incubated with primary antibodies against IL-17A (1:200), TRAF6 (1:300), p-ERK1/2 (1:150) or TNF-α (1:150) at 4°C overnight. The next day, sections were incubated with secondary antibody at room temperature for 1 hour. Immunoreactivity was localized with 3,30-diaminobenzide. Sections were observed and images were acquired using a BX53F optical microscope (Olympus, Tokyo, Japan) at 400× magnification. The integral optical density (IOD) was analyzed using Image J software (NIH, Bethesda, MD, USA) and the average optical density (AOD) was calculated using the ellipsoid formula below:


AOD=IOD/area

### Biochemical Analyses

Serum was collected as mentioned above. Serum levels of AST, ALT, creatinine and urea nitrogen were determined using an AU 480 Chemistry Analyzer (Beckman Coulter, Brea, CA, USA) according to the manufacturer’s instructions, separately.

### Statistical Analysis

Statistical analysis was performed using SPSS 20.0 software (IBM, Armonk, NY, USA). Data were presented as mean ± standard error of mean (SEM). Shapiro-Wilk test was used to perform normality test. One-way ANOVA test and non-parametric tests were used to determine the significance of data with normal or non-normal distribution separately. Difference were considered significant when *P*<0.05.

## Results

### JYKT Administration Reduced the Thyroid Volume

**Figure 3:**
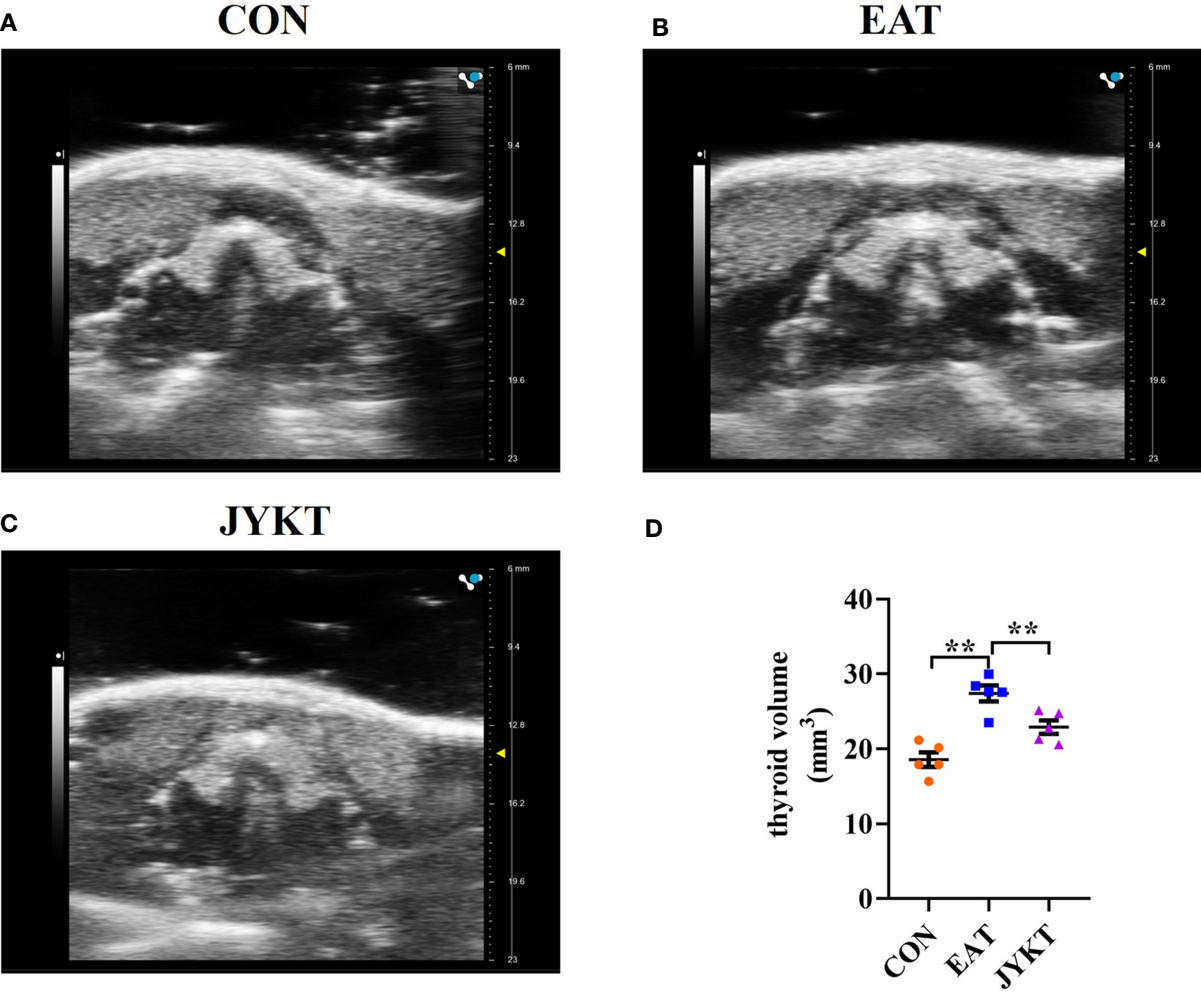
Morphology of thyroid in the CON **(A)**, EAT **(B)** and JYKT group **(C)** by ultrasonography. **(D)** Thyroid volumes among groups. ^**^ *P* < 0. 01.

Thyroid volume can change over the course of autoimmune thyroiditis and it reflects changes in function of the thyroid. To determine whether JYKT affects thyroid volume, the noninvasive, rapid and accurate ultrasonography was conducted. As shown in **Figure 3**, the horseshoe shaped thyroid lobes with high echogenicity were distinguished from surrounding tissues. Thyroids of the EAT group were significantly larger than those of the CON group (*P*<0.01). And compared with the EAT group, there was a significant reduction in the thyroid volume of the JYKT group (*P*<0.01). JYKT reduced mean thyroid volumes by 16.5%.

### JYKT Administration Resulted in Decreased Serum Levels of TPOAb and TGAb

**Figure 4:**
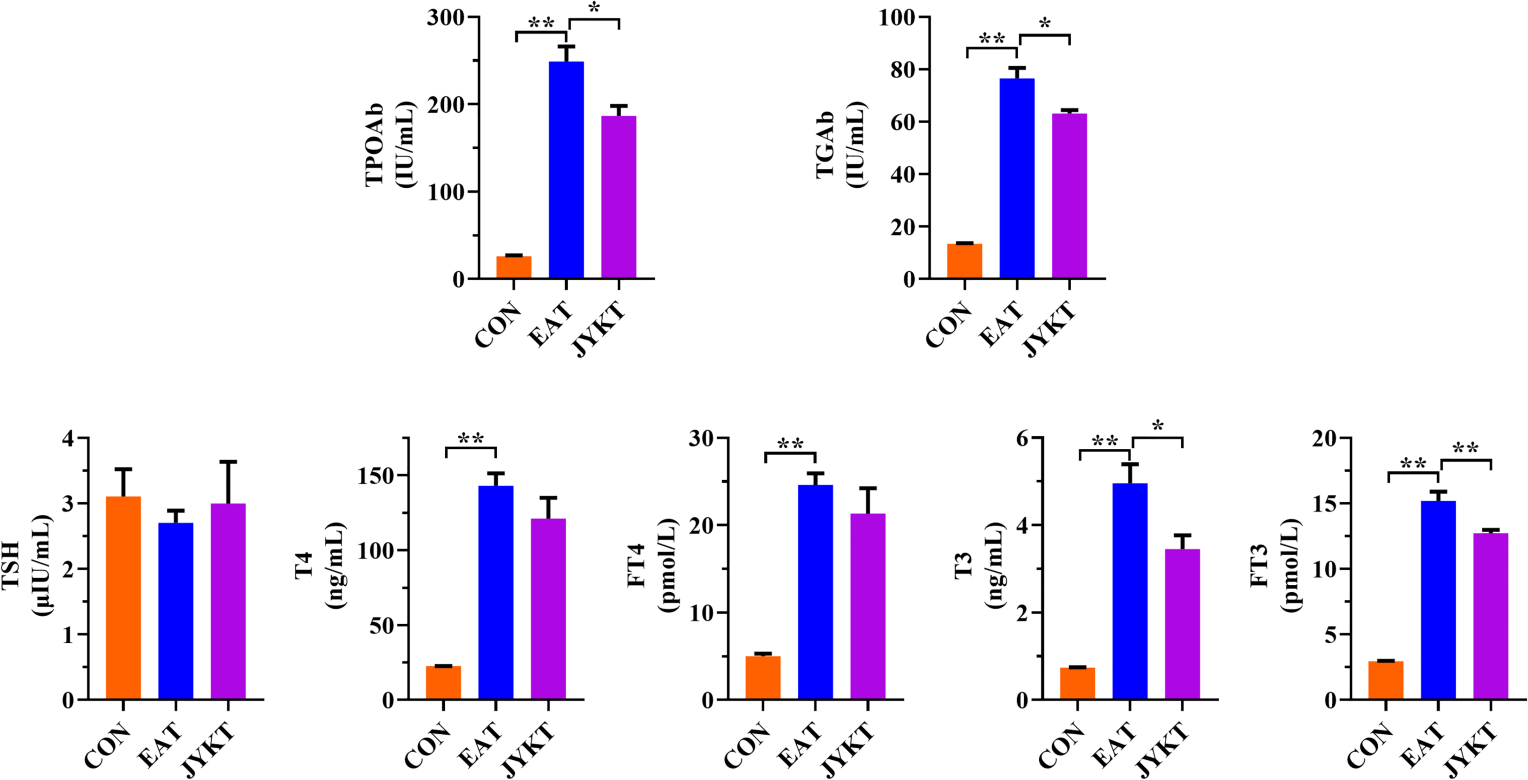
Serum levels of thyroid autoantibodies and hormones. ^**^ *P* < 0. 01, ^*^P < 0. 05.

Autoimmune thyroiditis is characterized by the development of autoantibodies, and excessive autoantibodies results in the destruction of the thyroid. To determine whether JYKT decreases the levels of autoantibodies, ELISA tests of TPOAb and TGAb were conducted. As shown in **Figure 4**, the average levels of TPOAb and TGAb of the EAT group were 9.5-fold and 5-fold, which were both significantly higher than those two average levels of the CON group (*P*<0.01). JYKT administration decreased both levels of TPOAb and TGAb (*P*<0.05).

To determine whether JYKT affects thyroid function, ELISA tests of TSH and thyroid hormones were conducted. As shown in **Figure 4**, the average levels of T4, FT4, T3 and FT3 of the EAT group and JYKT group were significantly higher than those of the CON group (*P*<0.01), as expected in this model of experimental autoimmune thyroiditis (18, 19). However, the average levels of T3 and FT3 were significantly lower in the JYKT group than in the EAT group (*P*<0.05). No significant differences of TSH levels were found among groups.

### JYKT Administration Alleviated Lymphocytic Infiltration of the Thyroid

**Figure 5:**
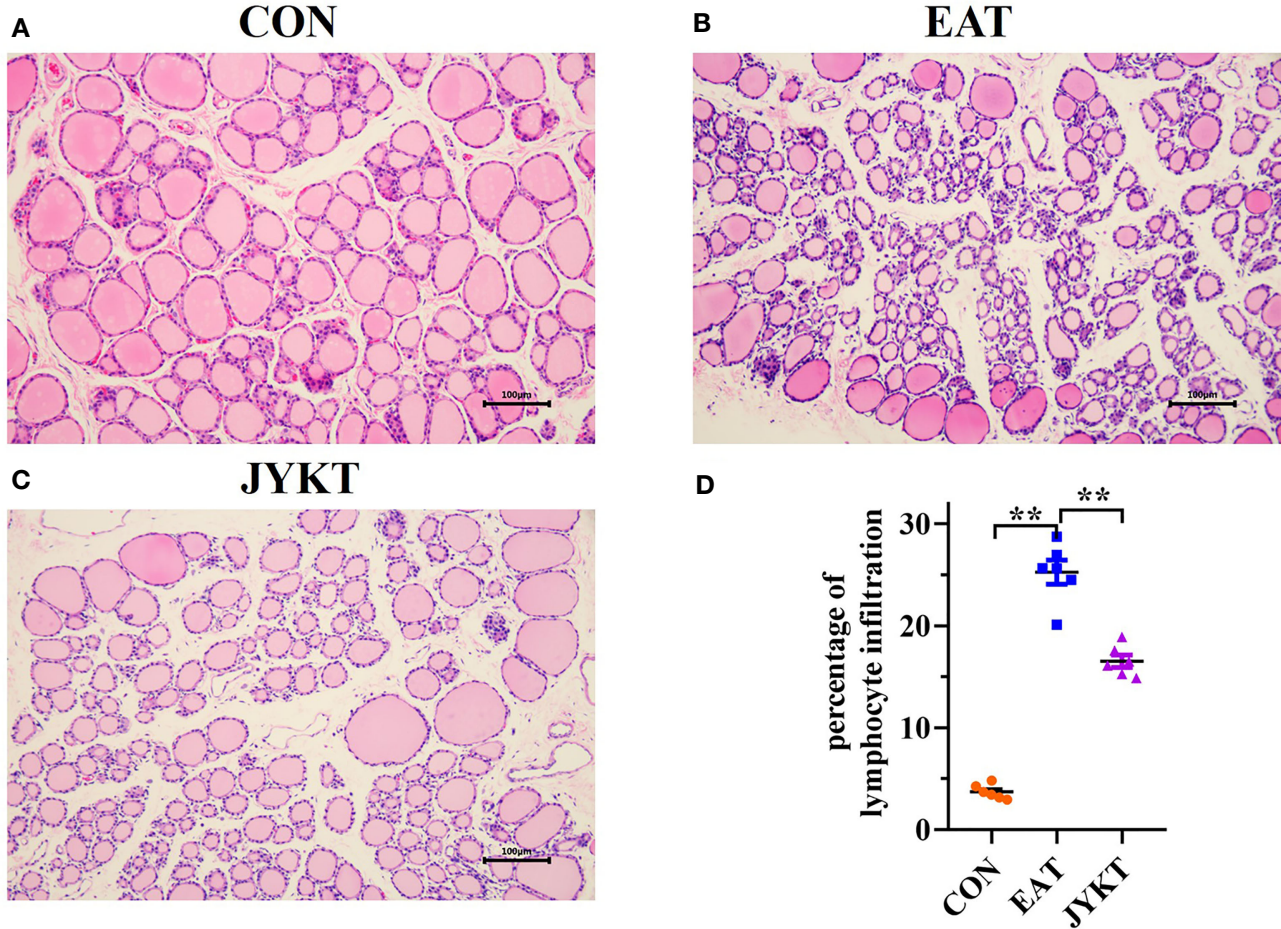
Pathology of thyroid in the CON **(A)**, EAT **(B)** and JYKT group **(C)** by H&E staining (200×). **(D)** Percentages of lymphocytic infiltration area among groups. ^**^ *P* < 0. 01.

Autoimmune thyroiditis is characterized by lymphocytic infiltration in the thyroid. To determine whether JYKT alleviates lymphocytic infiltration of the thyroid, H&E staining of sections was conducted, morphological changes of thyroid were observed, and percentages of lymphocytic infiltration area were analyzed. As shown in **Figure 5**, thyroid follicles of the CON group were moderately sized with a ring of epithelial cells, and pink-staining colloid inside was abundant with homogeneous distribution. In contrast, thyroid follicles of the EAT group were atrophied and abnormally shaped, with extensive lymphocytic infiltration. Colloid was inhomogeneous with atrophy. The percentages of lymphocytic infiltration area of the EAT group were significantly larger than those of the CON group (*P*<0.01). And compared with the EAT group, there was a significant reduction in the lymphocytic infiltration area of the JYKT group (*P*<0.01). JYKT alleviated lymphocytic infiltration of the thyroid.

### JYKT Administration Altered Metabolomics Profiles, and IL-17 Signaling Was Proved as a Critical Pathway in JYKT Administration for Autoimmune Thyroiditis

**Figure 6:**
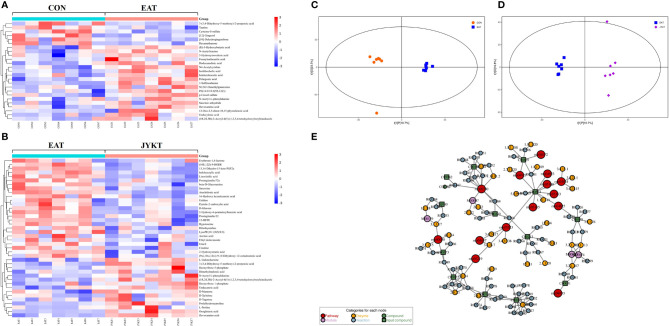
Heatmaps of differential metabolites between the CON and EAT group **(A)**, and between the EAT and JYKT group **(B).** OPLS-DA score plots between the CON and EAT group **(C)**, and between the EAT and JYKT group **(D)**. Network analysis between the EAT and JYKT group **(E)**.

To comprehensively identify metabolomics profiles among groups, differential metabolites with VIP>1 and *P*<0.05 were analyzed by OPLS-DA and visualized by clustering heatmaps. The heatmaps in **Figures 6A, B** showed upregulated and downregulated metabolites among groups. Upregulated metabolites are shown in red color, while downregulated metabolites in blue. A total of 25 differential metabolites between the CON and the EAT group were identified, and 40 between the EAT and the JYKT group were identified. Moreover, CON samples clustered on the left, whereas EAT samples clustered on the right in the OPLS-DA score plot in **Figure 6C**, showing significant differences in the metabolomic profiles between the CON and EAT group. There were also significant differences in the metabolomic profiles between the EAT and JYKT group in **Figure 6D**, indicating that these metabolites are associated with the effects of JYKT on autoimmune thyroiditis.

Metabolites and pathways are connected through intermediate reactions, enzymes and modules. To further determine how JYKT regulates metabolic network, pathway enrichment and network analysis between the EAT and JYKT group were combined. As shown in **Figure 6E**, a total of 14 pathways, 4 modules, 38 enzymes and 74 reactions were involved in the network. The 14 pathways were pentose phosphate pathway, arginine and proline metabolism, phospholipase D signaling pathway, C-type lectin receptor signaling pathway, IL-17 signaling pathway, oxytocin signaling pathway, regulation of lipolysis in adipocytes, renin secretion, AGE-RAGE signaling pathway in diabetic complications, Leishmaniasis, human cytomegalovirus infection, human papillomavirus infection, bladder cancer and rheumatoid arthritis. Interestingly, IL-17 signaling pathway (KEGG ID: 00030) was closely involved in the immunopathology of autoimmune thyroiditis among these pathways, suggesting that IL-17, the major effector cytokine of Th17 cells, may play a critical role in JYKT administration for autoimmune thyroiditis.

### JYKT Administration Downregulated Expressions of IL-17A, TRAF6, pERK1/2 and TNF-α

**Figure 7:**
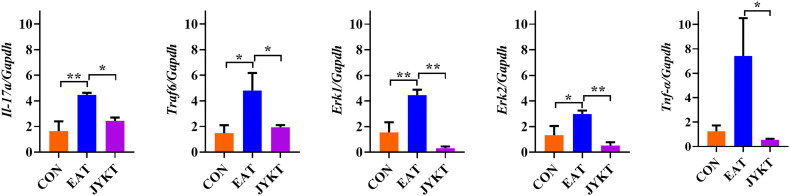
Relative gene expressions of *Il-17a, Traf6, Erk1, Erk2* and *Tnf-α* among groups. Data were shown as the relative expression ratio of targeted genes and *Gapdh*. ^*^ *P* < 0. 05, ^**^ *P* < 0. 01.

**Figure 8:**
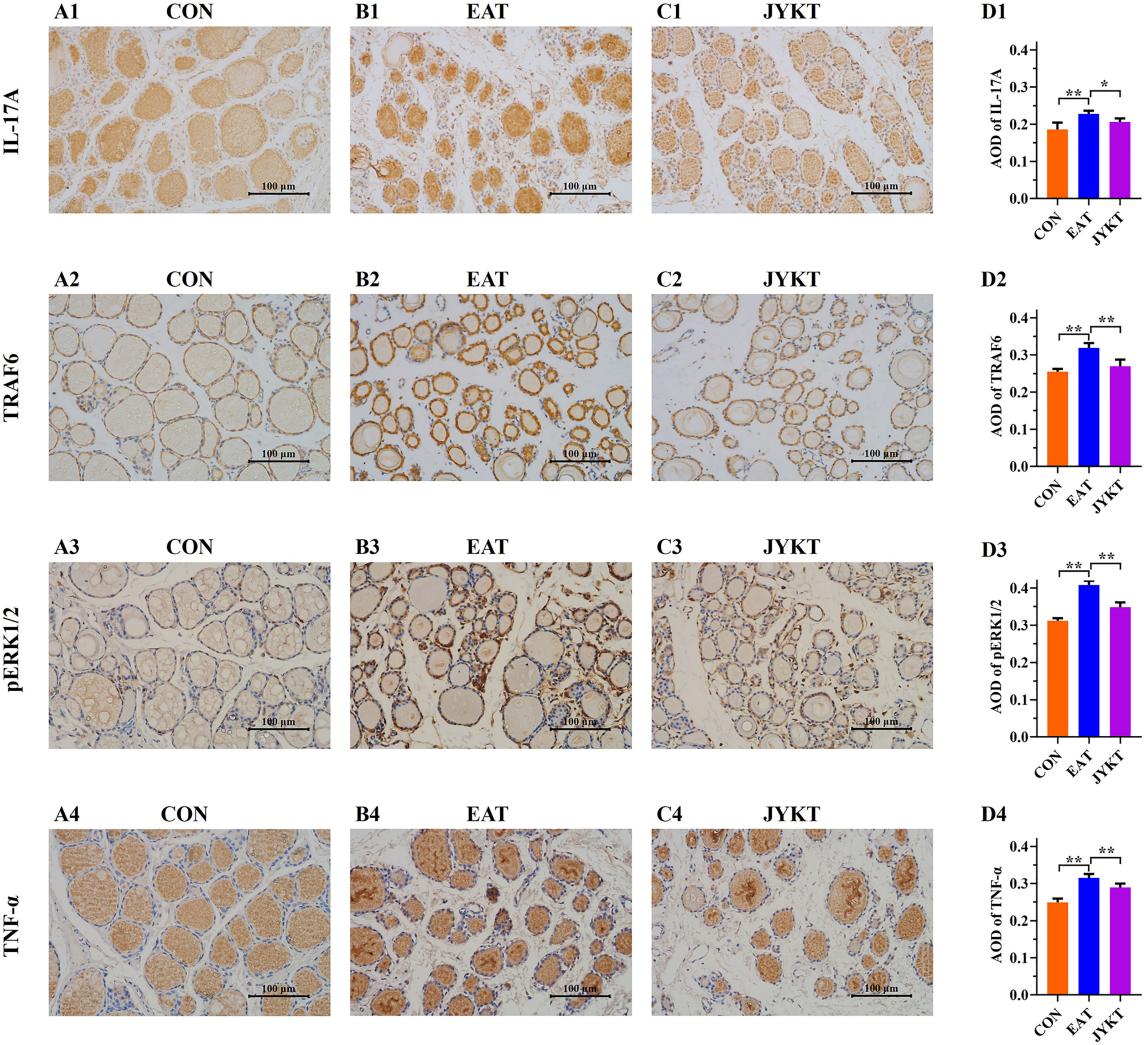
Immunoreactivity of IL-17A, TRAF6, pERK1/2 and TNF-α of thyroid in the CON **(A1–4)**, EAT **(B1–4)** and JYKT group **(C1–4)** (400×). Average optical densities among groups **(D1–4)**. ^**^ *P* < 0. 01, ^*^ *P* < 0. 05.

To further investigate how JYKT regulates IL-17 signaling, PCR of target genes and IHC of target proteins were conducted subsequently. As shown in **Figures 7** and **8**, expressions of genes and proteins of IL-17A, TRAF6, pERK1/2 and TNF-α in the JYKT group were significantly lower than those in the EAT group (**Figure 7**, *Il-17a*, *P*=0.023; *Traf6*, *P*=0.027; *Erk1* and *Erk2*, *P*<0.01; *Tnf-α*, *P*=0.043. **Figure 8**, IL-17A, *P*=0.010; TRAF6, pERK1/2 and TNF-α, *P*<0.01). Collectively, these results suggested that JYKT administration downregulated expressions of IL-17A, TRAF6, pERK1/2 and TNF-α.

### JYKT Administration Did Not Impair Functions of the Liver and the Kidney

AST and ALT are used to evaluate liver function, and creatinine in combination with urea nitrogen is used to evaluate renal function in clinics. To determine whether JYKT impairs functions of the liver and the kidney, serum levels of AST, ALT, creatinine and urea were determined. There were no significances of average levels of AST, ALT, creatinine and urea among groups (see **Supplementary Figure 1**). JYKT administration (2.834 g/kg per day) did not impair functions of the liver and the kidney in EAT rats.

## Discussions

AITDs are the most frequent T lymphocytes-mediated organ-specific autoimmune diseases and are considered to be associated with other autoimmune disorders and with papillary thyroid cancer as well (20, 21).

In this study, EAT was induced by immunizing rats with thyroglobulin and Freund’s adjuvant. Purified thyroglobulin with CFA was firstly injected subcutaneously to induce production of thyroglobulin-specific autoantibodies and inflammation of the thyroid, and thyroglobulin with IFA was successively injected to give rise to autoantibodies and enhance immune response (22). This is a widely-used *in vivo* model for studying the pathogenesis of autoimmune thyroiditis, characterized by infiltration of lymphocytes, destruction of thyroid follicles, and the development of autoantibodies (22–24). Emulsification of PTg in Freund’s adjuvant is a potent adjuvant, allowing a continuous delivery of PTg in a highly immunostimulatory environment. After immunization by PTg in Freund’s adjuvant, thyroid lesions can generally be detectable in one week, reach maximum in a month and maintain maximal state for two months, and finally, the chronic lesions last for at least 18 months (25). Therefore, the potent and stable modelling method was chosen in our study. By conducting ELISA tests, we found that the levels of TPOAb and TGAb of the EAT group were significantly higher than those of the CON group. By observing morphological changes and analyzing the percentage of lymphocytic infiltration area of H&E-stained sections, we found obvious lymphocytic infiltration in the EAT group, which was in accordance with previous studies.

T cells play indispensable role in modulating immune response, and dysfunction of T cells is closely associated with the pathogenesis of AITDs. Naive CD4^+^ T cells can differentiate into diverse subsets, maintaining the immune homeostasis. However, genetic susceptibility, environmental factors, drugs and psychologic stress can trigger dysfunction of the T cell subsets, resulting in immune attack against the thyroid and occurrence of AITDs (21). Recent studies on thyroiditis have focused on the imbalance between Th17 and Treg, and between Th1 and Th2 cells. Treg cells can suppress immune responses. Th17 cells, characterized by secretions of IL-17A and IL-23, are necessary to the removing of extracellular bacteria and maintaining of tissue homeostasis, and plays an essential role in the pathogenesis of AITD (26). Although the regulations and interactions of T cells in the pathogenesis of AITD are not totally elucidated, it is strongly believed that targeting Th17 cells and IL-17 can be a promising therapeutic strategy for AITDs (26). Therefore, we investigated the therapeutic effects of JYKT by targeting IL-17 signaling in an EAT rat model, aiming to provide a better understanding of JYKT administration for AITDs.

IL-17, the major effector cytokine of Th17 cells, participates in chronic inflammation and thyroid damages. After combined with IL-17R, IL-17A motivates MAPKs signaling, recruits macrophages and neutrophils, and results in proliferation of T cells and inflammation of the thyroid. Previous studies demonstrated that the level of IL-17A was significantly increased and the proportion of Th17 cells was significantly elevated in HT patients compared with the healthy controls (27–30). In this study, the expressions of IL-17A and downstream signaling were determined in rats with autoimmune thyroiditis. Consistent with the network analysis of metabolomics, we found increased expressions of IL-17A, TRAF6, ERK1/2 and TNF-α in the EAT group, compared with the CON group. And decreased expressions of these in the JYKT group were found, compared with the EAT group, indicating that IL-17 signaling plays a critical role in JYKT administration for autoimmune thyroiditis.

In the theory of TCM, the thyroid is located in the neck along the Liver meridian, and Liver Qi stagnation is responsible for the pathology of AITDs (31). TCM employs a holistic approach as therapeutic strategy for AITDs. JYKT, consists of nine Chinese herbs, has the effects of soothing Liver Qi, relieving depression, resolving phlegm and removing blood stasis. JYKT is proved to relieve symptoms, reduce thyroid size and decrease autoimmune antibody levels in patients with Hashimoto’s thyroiditis (13, 14). In this study, our data suggested that oral administration of JYKT at a dosage of 2.834 g/kg per day for eight weeks resulted in a reduction of thyroid volume and autoantibody levels, and alleviation of thyroid pathological changes in EAT rats. Metabolic network analysis proved IL-17 signaling pathway as a critical pathway in JYKT administration for autoimmune thyroiditis. Therefore, IL-17 signaling was conducted by PCR and IHC separately. The expressions of IL-17A, TRAF6, p-ERK1/2 and TNF-α were downregulated by JYKT administration. To the best of our knowledge, this is the first to discuss the effects of alternative medicine on regulating the signaling of IL-17, the major effector cytokine of Th17 cells, in an EAT rat model.

## Conclusion

This study demonstrated that JYKT alleviated inflammatory lesions in an EAT rat model by regulating IL-17 signaling. It provided experimental evidence supporting JYKT as a promising alternative therapy for autoimmune thyroiditis. Further studies are required to elucidate mechanisms of JYKT, and explore the combination of JYKT and modern therapy for AITDs.

## Data Availability Statement

The original contributions presented in the study are included in the article/**Supplementary Material**, further inquiries can be directed to the corresponding authors.

## Ethics Statement

The animal study was reviewed and approved by Animal Care and Use Committee of Beijing University of Chinese Medicine.

## Author Contributions

TL and KH conceived and designed the study. YP, QZ, and CZ performed the experiments. YP, LW, and LQ analyzed the data. YP wrote the manuscript. All authors contributed to manuscript read and approved the submission.

## Funding

The study is supported and funded by a Development of Science and Technology Project of Beijing University of Chinese Medicine: *The mechanisms of effects of JiaYanKangTai on experimental autoimmune thyroiditis* (No. 2021-ZXFZJJ-039).

## Conflict of Interest

The authors declare that the research was conducted in the absence of any commercial or financial relationships that could be construed as a potential conflict of interest.

## Publisher’s Note

All claims expressed in this article are solely those of the authors and do not necessarily represent those of their affiliated organizations, or those of the publisher, the editors and the reviewers. Any product that may be evaluated in this article, or claim that may be made by its manufacturer, is not guaranteed or endorsed by the publisher.
