# Supplementary figures and images for: A Chinese Patent Medicine JiaYanKangTai Alleviates Inflammatory Lesions of Experimental Autoimmune Thyroiditis by Regulating Interleukin-17 Signaling
Source: Front Endocrinol (Lausanne). 2022 Jan 31;12:794568. doi: 10.3389/fendo.2021.794568 (PMC8843746; doi:10.3389/fendo.2021.794568)

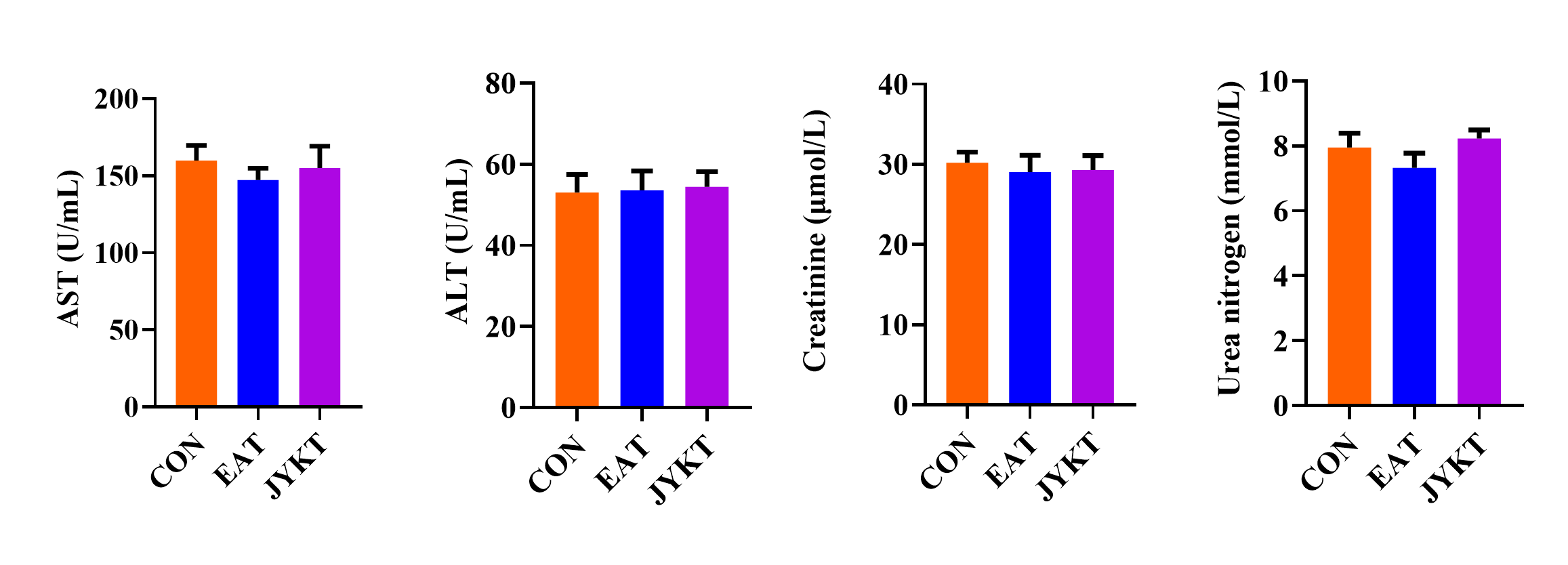

Supplement: Supplementary file 1 [file Image_1.tif]
